# Sense of Coherence or resilience as predictors of psychological distress in nursing students during the COVID-19 pandemic

**DOI:** 10.3389/fpubh.2023.1233298

**Published:** 2023-08-17

**Authors:** Leila Hasimi, Mehrnaz Ahmadi, Shahla Assadi Hovyzian, Ali Ahmadi

**Affiliations:** ^1^Department of Nursing, School of Nursing and Midwifery, Social Determinants of Health Research Center, Ahvaz Jundishapur University of Medical Sciences, Ahvaz, Iran; ^2^Medical and Surgical Nursing Department, School of Nursing and Midwifery, Social Determinants of Health Research Center, Ahvaz Jundishapur University of Medical Sciences, Ahvaz, Iran; ^3^Medical and Surgical Nursing Department, School of Nursing and Midwifery, Abadan University of Medical Sciences, Abadan, Iran; ^4^Department of Nursing, Student Research Committee, Ahvaz Jundishapur University of Medical Sciences, Ahvaz, Iran

**Keywords:** Sense of Coherence, academic resilience, psychological distress, nursing students, COVID-19

## Abstract

**Background:**

The COVID-19 pandemic lead to the occurrence of numerous psychological distress among students. This study aimed to determine the level of psychological distress as well as the predictive role of Sense of Coherence (SOC) and resilience in nursing students.

**Methods:**

A cross-sectional descriptive study was conducted on 310 nursing students in Ahvaz Jundishapur University of Medical Sciences, Iran. The data of the study was collected through the demographic information questionnaire, the General Health Questionnaire (GHQ), the Academic Resilience Inventory (ARI), and the Sense of Coherence Scale (SOC-13).

**Results:**

Students' mean scores for the GHQ, ARI, and SOC were 5.81 ± 1.37, 102.88 ± 11.91, and 54.54 ± 6.46, respectively. Regression models showed that two domains of SOC [meaningfulness (β = −0.28, *p* < 0.001), manageability (β = −0.19, *p* = 0.001)], female gender (β = 0.12, *p* = 0.015), and overall ARI (β = −0.12, *p* = 0.037), were significantly associated with the GH of nursing students. SOC domains [meaningfulness (β = −0.19, *p* = 0.002), manageability (β = −0.15, *p* = 0.006)], problem-oriented/positive thinking domain of ARI (β = −0.15, *p* = 0.011), sex (β = 0.12, *p* = 0.015), and history of death in first-degree relatives (β = 0.12, *p* = 0.021) were significantly associated with social dysfunction domain of GH. Three domains of SOC [meaningfulness (β = −0.26, *p* < 0.001), manageability (β = −0.13, *p* = 0.032), and comprehensibility (β = −0.13, *p* = 0.039)], were significantly associated with psychological distress domain of GH.

**Conclusion:**

Our results indicated that low SOC and resilience were predictors of psychological distress in nursing students. Accordingly, interventions such as teaching stress management skills, the skills of using positive coping methods in dealing with stressful situations, and self-management skills are necessary to improve the level of resilience and SOC in nursing students.

## Introduction

The COVID-19 pandemic not only resulted in widespread public health concerns but also led to the emergence of several psychological conditions among individuals, particularly students (1–3). Because of the pandemic, universities were closed and the classes were held virtually, which led to significant levels of anxiety and stress among students (3, 4). Similar to nurses in other regions of the world, nursing students in Iran were also involved in caring for COVID-19 patients, which placed a significant mental strain on these students (2). Fear of disease, fear of death, witnessing the death of patients, and inadequate clinical competence to provide care to patients, on the one hand (5, 6), and worrying about the effect of this virus on the educational and professional future of the students, on the other, lead to stress, anxiety, sadness, grief and overall psychological distress in these students (5–7).

Psychological distress is defined as states of apathy, depression, anxiety and hopelessness about the future (8). Many studies have hitherto investigated the prevalence of psychological disorders in students during the COVID-19 pandemic. Based on the results of these studies, this disease has had destructive psychological effects on the mental health of students, especially medical students. According to studies, medical students have experienced higher levels of depression, anxiety, and stress during the pandemic, as the level of these disorders in medical students has been reported to be 3–8.7% higher than ordinary people (9). In a systematic review that reviewed 27 studies in the area of mental health of students during the COVID-19 pandemic, 14 mental health problems were identified in students, of which depression and anxiety had the highest prevalence of 39 and 36%, respectively (10). Studies conducted in Iran also show that nursing students suffer from many psychological disorders, including anxiety, during the pandemic (11).

Psychological distress can cause extensive changes in one's daily relationships and social interactions (8). Therefore, we need more investigations to identify its dimensions in students and detect the protective factors affecting it. As indicated in several studies, efficient utilization of psychological coping mechanisms, including Sense of Coherence (SOC) and resilience, can help people manage stressful events as these mechanisms play a substantial role in preventing mental disorders (12–14). According to the results of a study in Iran, SOC and resilience play a crucial role in reducing the COVID-19-related anxiety in students and, hence, targeting these components through psychological therapeutic actions effectively reduce this type of anxiety in students (13).

SOC is defined as one's ability to recognize life's stressors and, then, use coping resources efficiently. A strong internal SOC provides people with more power to deal with anxiety-provoking life experiences and helps them pass through the stages of life with higher strength (15). In critical situations such as the COVID-19 pandemic, when people's mental health is endangered, high SOC can help control mental problems better and more effectively and endow people with higher personal wellbeing (16).

Moreover, some characteristics and skills such as resilience can increase the ability of students in dealing with difficult situations and overcoming life and academic challenges. Resilience refers to one's ability to successfully adapt to difficult conditions, and reduces one's vulnerability to psychological disorders and emotional disturbances (17). The function of resilience mechanism in reducing psychological distress is such that its main components, including self-confidence, trust in instincts, personal competence, and control, act as a buffer when a person is placed in stressful situations and does not allow psychological distresses to occur (18). Academic resilience has been identified as a coping method for educational challenges (19) and defined as students' ability to overcome academic stress or pressure (50). For nursing students, who must engage in learning and clinical practice, academic resilience is a protective factor for coping with academic stress and helps them better adjust to their academic environment and the clinical setting and leads them to play professional roles in the future (20, 21). Studies have emphasized the role of resilience as a mediator in the relationship between the COVID-19-related stress and psychological wellbeing in nursing students (22). Additionally, based on studies, nursing students with higher resilience have better academic performance, better mental health and lower academic burnout (19, 23, 50).

In achieving success in the fight against epidemics such as the coronavirus, it is necessary to assess the mental health of students who play a role in the field of health and care and adjust their mental stress and support them. Although several studies have been carried out in Iran in the field of mental health, resilience, and SOC in nursing students during the COVID-19 pandemic, however, a few studies have been conducted in the field of academic resilience and its relationship with the SOC as well as the role of these two variables in predicting psychological distress in nursing students. Given the significance of the mental health of nursing students and their inherently stressful job, investigation of effective coping mechanisms such as academic resilience and a SOC can be useful for designing intervention programs to empower nursing students and can lead to the creation of holistic views for preparing these students for similar situations in the future. Therefore, the present study was conducted to determine the level of psychological distress as well as the predictive role of SOC and resilience in nursing students of Ahvaz Jundishapur University of Medical Sciences during the COVID-19 pandemic.

## Design and sample

This study was a cross-sectional and descriptive-correlational study conducted in the faculty of nursing at Ahvaz Jundishapur University of medical sciences, Ahvaz, Iran. The data were collected from October 2021 to December 2021 through convenience sampling with a population of undergraduate nursing students using an online questionnaire. An informed consent form (with an explanation describing the purpose of the study, procedures, and the rights of participants) and a link to the Google survey were sent through social networks. The sample size was determined considering the size of the society (600 people). Two hundred and thirty-five (235) nursing students were selected using the Cochrane statistical formula (95% confidence interval and 80% power assessment). This online survey was sent to 360 students to allow for possible variability, and 310 students responded. The response rate was 86.11%.

## Measurements

### Demographic information questionnaire

A demographic information questionnaire included the students' age, sex, marital status, academic year, history of infection with COVID-19 (yes and no), and history of death due to infection with COVID-19 in first-degree relatives (yes and no).

### General Health Questionnaire

We use the Iranian version of the GHQ-12. This questionnaire has 12 questions and two dimensions of social dysfunction and psychological distress. A 0-0-1-1 bimodal scoring method is used for the 12 questions (0 = absence of common mental disorders, 1 = presence). Overall scores range from 0 to 12, with higher scores indicating worse conditions. A GHQ-12 score of 4 or higher indicated psychological distress. The validity and reliability of the Persian version of the GHQ have been evaluated by Najarkolaei et al. (24). In this study, the reliability of the scale using Cronbach's alpha coefficient (0.7) was satisfactory.

### Academic Resilience Inventory

The ARI was developed by Samuels et al. (25) as a valid and reliable scale in 2009. The Persian version of ARI has been psychometrically evaluated in Iran by Soltaninejad et al. (26), with Cronbach's alpha level of 0.62-0.76. In the present study, the internal consistency of ARI was evaluated and confirmed with Cronbach's alpha of 0.80. The Iranian version of ARI contains 29 questions and three subscales: communication skills (13 items), orientation toward the future (10 items), and problem-oriented/positive thinking (six items). The students' responses were rated on a 5-point Likert scale ranging from 1 (completely disagree) to 5 (completely agree). The overall score of the scale varies from 29 to 145, which is a higher score indicating better resilience in students.

### Sense of Coherence scale

Nursing student SOC was measured using an abbreviated form of the SOC scale. It was developed by Antonovsky (15) and consists of 13 items with three subscales: comprehensibility (five items), manageability (four items), and meaningfulness (four items). An individual's responses are rated on her 7-point semantic scale from 1 to 7. Overall scores range from 13 to 91, with higher scores indicating stronger SOC (15). The validity and reliability of the Persian version of SOC have been validated in Iran by Rohani et al. evaluated (27). In this study, the reliability of the scale using Cronbach's alpha coefficient (0.72) was satisfactory.

### Data analysis

Data analysis was performed using SPSS v.22 software. Since the Kolmogorov-Smirnov test showed a normal distribution of the data, parametric tests were used. Independent *t*-test and one-way ANOVA were used to compare mean GHQ, resilience and SOC across dichotomous demographic variables. Significant population groups, resilience, and SOC were entered into the regression model as independent variables. Relationships between outcome variables (GHQ, SOC, and resilience) were evaluated in correlation matrices using Pearson's correlation coefficients before performing regression analysis. A stepwise multiple regression analysis was performed to determine predictors of psychological distress in nursing students (*P*-value for entry < 0.05).

### Ethical considerations

This research has been approved by the Ethics Committee of Ahvaz Jundishapur University of Medical Sciences (ethics code: IR.AJUMS.REC.1400.474). Ethical considerations such as obtaining approval from the relevant authorities, explaining the purpose of the study to participants and ensuring data confidentiality, and obtaining verbal consent from samples were adhered to.

## Results

A total of 310 nursing students were included in the study. The mean age of the students was 21.98 ± 2.86 years. Most of them (67.8%) were female, 36.8% of students had a history of the COVID-19 infection and 58% had a history of death due to infection with COVID-19 in first-degree relatives. Demographic characteristics of the students according to the mean levels of their General Health (GH), Academic Resilience (AR), and SOC are presented in Table 1. It shows that there were no significant differences between demographic categories in terms of students' mean GH scores, except for the variables of sex, and history of death due to infection with COVID-19 in first-degree relatives in total GH and the dimension of social dysfunction (*p* < 0.05). No significant differences were found between demographic categories in terms of students' mean SOC and AR. However, a significant difference was found between the dimension of Manageability of SOC and sex (*p* = 0.02), as well as between the dimension of future orientation of AR and history of death due to infection with COVID-19 in first-degree relatives (*p* = 0.04; Table 1).

**Table 1:** Demographic variables of nursing students based on three outcome variables; the GHQ, the ARI, and the SOC (*n* = 310).

**Variables**		**Number (%)**	**Psychological distress (M ±SD)**	**Social dysfunction (M ±SD)**	**Total GHQ (M ±SD)**	**Total ARI (M ±SD)**	**Total SOC (M ±SD)**
Age	18–20	151 (48.7)	3.03 ± 0.82	2.89 ± 0.90	5.92 ± 1.42	103.94 ± 10.80	54.07 ± 6.19
	≤ 21	159 (51.3)	2.94 ± 0.84	2.76 ± 0.89	5.71 ± 1.32	101.87 ± 12.83	54.99 ± 6.693
	*p*-value		0.38	0.21	0.17	0.12	0.21
Sex	Male	100 (32.2)	2.93 ± 0.90	2.68 ± 0.89	5.62 ± 1.49	101.52 ± 13.10	54.53 ± 6.06
	Female	210 (67.8)	3.01 ± 0.80	3.29 ± 0.90	6.50 ± 1.21	103.51 ± 11.31	54.59 ± 6.65
	*p*-value		0.46	0.01	0.01	0.19	0.94
Academic year	First-year	88 (28.4)	3.04 ± 0.88	2.87 ± 0.91	5.92 ± 1.45	104.17 ± 11.22	54.09 ± 6.40
	Second-year	47 (15.2)	2.95 ± 0.95	2.82 ± 0.96	5.78 ± 1.61	101.89 ± 12.86	53.85 ± 6.11
	Third-year	112 (36.1)	2.91 ± 0.82	2.68 ± 0.90	5.59 ± 1.40	102.41 ± 11.86	54.28 ± 6.52
	Fourth-year	63 (20.3)	3.07 ± 0.70	3.01 ± 0.79	6.09 ± 0.89	102.65 ± 12.34	56.17 ± 6.57
	*p*-value		0.53	0.12	0.11	0.67	0.16
History of infection with COVID-19	Yes	114 (36.8)	2.91 ± 0.91	2.73 ± 1	5.64 ± 1.52	103.14 ± 12.13	55.16 ± 6.91
	No	196 (63.2)	3.03 ± 0.78	2.88 ± 0.82	5.91 ± 1.27	102.73 ± 11.81	54.18 ± 6.17
	*p*-value		0.21	0.16	0.09	0.77	0.2
History of death due to infection with COVID-19 in first-degree relatives	Yes	180 (58)	2.97 ± 0.88	2.73 ± 0.92	5.70 ± 1.38	103.50 ± 11.71	54.94 ± 6.58
	No	130 (42)	3 ± 0.77	2.96 ± 0.857	6.42 ± 1.74	102.17 ± 12.12	54.05 ± 6.27
	*p*-value		0.71	0.01	0.02	0.33	0.22

GHQ, General Health Questionnaire; ARI, Academic Resilience Inventory; SOC, Sense of Coherence.

The mean of the GH, AR, and SOC were 5.81 ± 1.37, 102.88 ± 11.91, and 54.54 ± 6.46, respectively. In addition, a total of 293 (94.5%) students showed psychological distress (Table 2).

**Table 2:** Descriptive data for outcome variables, including the GHQ, ARI, and the SOC in nursing students (*n* = 310).

**Outcome variables**	**Mean ±SD**	**Range in the scales**
Total GHQ	5.81 ± 1.37	0–12
Psychological distress	2.99 ± 0.83	0–6
Social dysfunction	2.82 ± 0.89	0–6
Total ARI	102.88 ± 11.91	29–145
Communication skills	47.08 ± 47.08	13–65
Future orientation	35.35 ± 4.32	10–50
Problem-oriented/positive orientation	20.44 ± 3.45	6–30
Total SOC	54.54 ± 6.46	13–91
Meaningfulness	19.05 ± 4.22	4–28
Manageability	15.34 ± 4.15	4–28
Comprehensibility	19 ± 4.54	5–35

GHQ, General Health Questionnaire; ARI, Academic Resilience Inventory; SOC, Sense of Coherence.

### Regression analyses results

A correlation matrix was created as a prerequisite for regression analysis. A significant negative correlation was found between the GH score with the SOC (*r* = −0.16, *P* < 0.001), and the AR (*r* = −0.35, *P* < 0.001). A significant positive correlation was also found between SOC and AR (*r* = 0.19, *P* < 0.001; Table 3).

**Table 3:** Correlation matrix between variables of the GHQ, ARI, and the SOC in nursing students.

**Variables**	**Psychological distress**	**Social dysfunction**	**GHQ**	**Communication skills**	**Future orientation**	**Problem- oriented/ positive orientation**	**ARI**	**Meaningfulness**	**Manageability**	**Comprehensibility**	**SOC**
Psychological distress	1	0.350^**^	0.807^**^	−0.256^**^	−0.178^**^	−0.165^**^	−0.250^**^	−0.364^**^	−0.312^**^	−0.282^**^	−0.179^**^
Social dysfunction	0.350^**^	1	0.835^**^	−0.278^**^	−0.255^**^	−0.284^**^	−0.324^**^	−0.359^**^	−0.303^**^	−0.203^**^	−0.090
GHQ	0.807^**^	0.835^**^	1	−0.325^**^	−0.265^**^	−0.276^**^	−0.350^**^	−0.440^**^	−0.374^**^	−0.294^**^	−0.162^**^
Communication skills	−0.256^**^	−0.278^**^	−0.325^**^	1	0.518^**^	0.588^**^	0.896^**^	0.483^**^	0.362^**^	0.268^**^	0.194^**^
Future orientation	−0.178^**^	−0.255^**^	−0.265^**^	0.518^**^	1	0.527^**^	0.794^**^	0.484^**^	0.267^**^	0.233^**^	0.132^*^
Problem–oriented/positive orientation	−0.165^**^	−0.284^**^	−0.276^**^	0.588^**^	0.527^**^	1	0.797^**^	0.507^**^	0.192^**^	0.172^**^	0.147^**^
ARI	−0.250^**^	−0.324^**^	−0.350^**^	0.896^**^	0.794^**^	0.797^**^	1	0.582^**^	0.347^**^	0.279^**^	0.195^**^
Meaningfulness	−0.364^**^	−0.359^**^	−0.440^**^	0.483^**^	0.484^**^	0.507^**^	0.582^**^	1	0.419^**^	0.308^**^	0.153^**^
Manageability	−0.312^**^	−0.303^**^	−0.374^**^	0.362^**^	0.267^**^	0.192^**^	0.347^**^	0.419^**^	1	0.516^**^	0.350^**^
Comprehensibility	−0.282^**^	−0.203^**^	−0.294^**^	0.268^**^	0.233^**^	0.172^**^	0.279^**^	0.308^**^	0.516^**^	1	0.517^**^
SOC	−0.179^**^	−0.090	−0.162^**^	0.194^**^	0.132^*^	0.147^**^	0.195^**^	0.153^**^	0.350^**^	0.517^**^	1

According to Pearson correlation coefficients: ^*^p < 0.05; ^**^p < 0.001.

GHQ, General Health Questionnaire; ARI, Academic Resilience Inventory; SOC, Sense of Coherence.

Three multiple linear regression analyses were performed using the stepwise method to determine the predictors of GH, psychological distress, and social dysfunction in nursing students (Table 4). The results showed that two domains of SOC [the meaningfulness (β = −0.28, *p* < 0.001), and the manageability (β = −0.19, *p* = 0.001)], as well as sex (β = 0.12, *p* = 0.015), and overall ARI (β = −0.12, *p* = 0.037), were significantly associated with the GH of nursing students, explaining 26% of the variance (*F* = 26.73, *p* < 0.001). The meaningfulness domain (β = −0.19, *p* = 0.002), the manageability domain (β = −0.15, *p* = 0.006), the problem-oriented/positive thinking domain of ARI (β = −0.15, *p* = 0.011), sex (β = 0.12, *p* = 0.015), and history of death due to infection with COVID-19 in first-degree relatives (β = 0.12, *p* = 0.021), were significantly associated with social dysfunction domain of GH. The model explained 18% of the variance in this domain (*F* = 15.24, *p* < 0.001). Three domains of SOC [the meaningfulness (β = −0.26, *p* < 0.001), the manageability (β = −0.13, *p* = 0.032), and the comprehensibility (β = −0.13, *p* = 0.039)], were significantly associated with psychological distress domain of GH. The model explained 16% of the variance in this domain (*F* = 21.77, *p* < 0.001).

**Table 4:** Summary results of the multiple linear regression analyses with psychological distress, social dysfunction, and GHQ as dependent variables.

**Model**	***R***	***R* ^2^**	***AdjR* ^2^**	**Independent variables**	**B**	**SE**	**β**	***t***	***p***
GHQ	0.50	0.26	0.25	Constant	24.11	1.30	–	18.51	< 0.001
				Meaningfulness	−0.19	0.04	−0.28	−4.45	< 0.001
				Manageability	−0.13	0.03	−0.19	−3.45	< 0.001
				Sex	0.74	0.30	0.12	2.45	0.015
				ARI	−0.03	0.01	−0.12	−2.09	0.037
Social dysfunction	0.44	0.20	0.18	Constant	10.69	0.77	–	13.757	< 0.001
				Meaningfulness	−0.08	0.02	−0.19	−3.07	0.002
				Manageability	−0.06	0.02	−0.15	−2.76	0.006
				Problem–oriented/positive orientation	−0.07	0.03	−0.15	−2.55	0.011
				Sex	0.48	0.19	0.12	2.44	0.015
				History of death due to infection with COVID-19 in first–degree relatives	0.42	0.18	0.12	2.31	0.021
Psychological distress	0.41	0.17	0.16	Constant	12.444	0.470	–	26.026	< 0.001
				Meaningfulness	−0.106	0.02	−0.26	−4.65	< 0.001
				Comprehensibility	−0.048	0.02	−0.13	−2.14	0.032
				Manageability	−0.053	0.02	−0.13	−2.07	0.039

## Discussion

The purpose of this cross-sectional study was to determine the level of psychological distress, resilience, and SOC in a sample of nursing students, and to explore the predictor role of resilience and/or SOC for students' psychological distress. Our results showed that the level of psychological distress of students was average to high. It is not a surprising result as we can find the same results in earlier studies (5, 8, 22, 28, 50). Not only did the pandemic put people under the psychological pressure of its numerous peaks, but it also caused other problems such as the crises of knowledge, digital life, the fear of education's future, and the loss of friends and relatives. These crises jeopardized the mental health of the students and caused many changes in their personal and social relationships, moods, desires and needs. These mental pressures and crises together increased the rate of mental disorders such as depression, anxiety, worry and stress, and threatened the social growth and identity of the students. They also decreased social vitality of medical students, especially nursing students.

Furthermore, our sampling was performed when universities reopened after a long period of virtual education. This had also increased the stress and anxiety of the students and, generally, the students were experiencing high levels of psychological distress during that time. Thus, screening and psychological interventions together with the improvement of coping skills need to be prioritized in order to promote the mental health of nursing students.

In the present study, there was no significant difference in the GHQ scores of nursing students in terms of age, marital status, academic year, and history of infection with COVID-19. This result is in line with some previous studies (29–31). However, a significant difference was observed between male and female students in terms of the Social Dysfunction dimension and the total score, so that girls had higher psychological disorders than boys. In line with the present study, previous studies have also shown that the prevalence of mental disorders in women is higher than men (32, 33). The higher prevalence of mental disorders in women is perhaps due to the different way they deal with difficult and stressful situations, as well as hormonal fluctuations in women. Nonetheless, in a study conducted in Bangladesh, the prevalence of depression and anxiety in male students was higher than that of female students (34). Other studies have found no gender difference with regard to mental health (35). This contradiction is probably because of the difference in the cultures of the investigated countries and, thus, there is a need for more evaluations in this regard.

Despite the high levels of psychological distress reported by nursing students, their level of resilience and SOC was average. These results are consistent with the results of some previous studies (5, 19, 22, 28, 49). The reason is perhaps due to the fact that nursing students are educated about different coping methods and health-promoting behaviors. Therefore, compared to ordinary people, nursing students are more capable in using personal coping resources such as resilience and SOC and, consequently, they can deal with stressful situations more powerfully. However, compared to the studies conducted before the COVID-19 pandemic in the area of resilience and SOC of nursing students, the levels of these coping strategies have decreased relatively (36, 37). This suggests that although there are coping strategies, they are not adequate and, therefore, students could not cope with the stressful factors sufficiently. This issue can have serious consequences for the mental health of nursing students in the long run. Resilience is a psychological coping mechanism that causes a person to develop more flexibility and compromise in facing with the challenges of life, and acquire self-management skills and thoughts (18). SOC is also a way of thinking and acting with an inner confidence that reflects one's view of life and the capacity of responding to stressful situations. SOC helps people improve their mental health through appropriate use of coping mechanisms (38). Since the COVID-19 pandemic has had lasting effects on the psyche of people, including students, it is a big challenge to maintain and improve the level of resilience and SOC of students. Improved perception of students of stressful experiences can be assumed as an important coping strategy which can significantly promote the level of resilience and SOC in students (16, 36). Accordingly, there is a need to think about the measures and interventions which can improve the self-management skills of the nursing students and empower them in the optimal and efficient use of coping strategies in order to prepare them for dealing with similar situations in the future.

In examining SOC and resilience, no significant difference was observed in the total score or any of the dimensions in terms of age, gender, marital status, academic year, and history of the COVID-19 infection. These results are in line with some previous studies (39, 40).

However, based on a unique finding of our study, those students whose first-degree relatives had died of the COVID-19 had higher mental health in the total score and the Social Dysfunction dimension, as well as higher resilience in the dimension of the future orientation. This finding is not in line with the results of the studies conducted during the pandemic, indicating that the infection of relatives and acquaintances with COVID-19 was a risk factor for increasing anxiety in students (41). Resilience is not limited to overcoming loss and coping with painful experiences, but includes positive adaptation, that is, regaining one's ability after experiencing turmoil (42). Thus, it can be argued that people with the experience of losing their loved ones during the pandemic, adopt the coping strategies more optimally as they have developed more adaptation and positive growth resulting from this painful experience. As a result, they reported higher levels of resilience, which had in turn a positive effect on their mental health.

Based on the results of the present study, a negative correlation was obtained between the scores of GHQ, resilience and SOC, and the students with higher resilience and SOC enjoyed higher mental health. Moreover, as the results of linear regression analysis showed, SOC and resilience were predictors of mental health of nursing students. A strong SOC is associated with a better perception of mental health (43). The results of our study are in line with the results of previous studies, showing that people with weak SOC have a higher risk of mental health problems (14, 28, 44). SOC is a variable which moderates mental health status. It is believed that the effects of SOC on mental health work through two mechanisms; based on the first mechanism, people with stronger SOC perceive less stress in daily life and, according to the second one, SOC can moderate stressful factors. As such, effective stress management can help students improve their level of SOC, thereby promoting their mental health (43).

In line with the results of the present study regarding resilience, some studies have shown that students with high resilience experienced less anxiety during the COVID-19 outbreak and had a better mental health (13, 22). Based on the results of a systematic review, high levels of resilience and appropriate use of coping skills not only reduce stress levels in nursing students, but also improve their psychological wellbeing (45). On the contrary, increased levels of psychological problems have been attributed to insufficient levels of resilience during stressful events (14, 46, 49, 50). Wolff argues that resilience is identified with characteristics such as social ability, efficient problem solving, and purposefulness, and believes that these characteristics have a positive relationship with mental health, so that improved level of these characteristics promotes people's resilience which in turn can naturally improves their mental health (47). Therefore, enhancing the social ability of students in dealing with incidents and events and teaching them the appropriate use of problem-solving methods will effectively improve their resilience level and, consequently, their mental health (48).

## Limitations of the study

This study has some limitations. First, due to the nature of the cross-sectional study design, we were unable to examine causal relationships between study variables. Long-term study designs have therefore been proposed to measure dynamic structures such as resilience and SOC and assess how these structures change over time. Second, the use of self-reported scales can lead to response bias during responses. Finally, SOC and resilience could only predict 26% of changes in common health variables. Therefore, future research should consider other individual and organizational variables that may influence the mental health of nursing students.

## Conclusion

Based on the results of the present study, during the COVID-19 pandemic, students suffered from high psychological distress and had moderate levels of resilience and SOC. Our results also indicated that low SOC, female gender, and low resilience were the predictors of mental health disorders in nursing students. Accordingly, interventions such as teaching stress management skills, the skills of using positive coping methods in dealing with stressful situations, and self-management skills are necessary to improve the level of resilience and SOC in nursing students. To this end, the organizations (nursing schools and hospitals) as well as nursing faculty members play a substantial role in training and strengthening resilience and SOC in nursing students to improve their mental health.

## Implication

The result of this study brings new insight into the importance of resilience and SOC as a way for improving nursing student's mental health. In addition, it is important to note this point developing academic resilience is not only the responsibility of the nursing students and educators but also the organization, nursing schools, and clinical settings. Therefore, the inclusion of academic resilience in nursing undergraduate curriculum training is necessary. Moreover, the personal characteristics of nursing students, such as SOC, must be considered throughout their nursing education. Some interventions should be thought of to lead to improving the level of resilience and SOC in nursing students, for example, specific programs involving nursing students' introspection to improve SOC and resilience should be offered as short training sessions before they begin clinical training. Educational leaders and faculty should also provide the necessary foundations to create the right learning spaces in clinical settings that support the improvement of the level of resilience and SOC. Furthermore, future research on the effects of academic resilience and SOC in nursing students in the context of a global health crisis could make a significant contribution to nursing education and the nursing profession.

## Data availability statement

The raw data supporting the conclusions of this article will be made available by the authors, without undue reservation.

## Ethics statement

The studies involving humans were approved by Ethics Committee of Ahvaz Jundishapur University of Medical Sciences (ethics code: IR.AJUMS.REC.1400.474). The studies were conducted in accordance with the local legislation and institutional requirements. The participants provided their written informed consent to participate in this study. No potentially identifiable images or data are presented in this study.

## Author contributions

MA and LH contributed to the study's conception and design. Material preparation, data collection, and analysis were performed by MA, LH, AA, and SH. The first draft of the manuscript was written by MA and LH. MA as a principal investigator supervised the project. All authors commented on previous versions of the manuscript and read and approved the final manuscript.
